# Unique Method for Prognosis of Risk of Depressive Episodes Using Novel Measures to Model Uncertainty Under Data Privacy

**DOI:** 10.3390/e27020162

**Published:** 2025-02-03

**Authors:** Barbara Pękala, Dawid Kosior, Wojciech Rząsa, Katarzyna Garwol, Janusz Czuma

**Affiliations:** 1 Institute of Computer Science, University of Rzeszów, 35-310 Rzeszów, Poland; dkosior@ur.edu.pl (D.K.); wrzasa@ur.edu.pl (W.R.); kgarwol@ur.edu.pl (K.G.); 2 Department of Artificial Intelligence, University of Information Technology and Management, 35-225 Rzeszów, Poland; 3 LUX MED—PROFEMED Medical Center, 35-315 Rzeszów, Poland; ddcad55@gmail.com

**Keywords:** interval-valued fuzzy set theory, interval-valued entropy, data uncertainty, federated decision-making, depression risk detection

## Abstract

The research described in this paper focuses on key aspects of learning from data concerning the symptoms of depression and how to prevent it. The computer support system designed for that purpose combines data privacy protection from various sources and uncertainty modeling, especially for incomplete data. The mentioned aspects are key to real-life medical diagnostic problems. From among the different paradigms of machine learning, a federated learning-based approach was chosen as the most suitable to take up the challenge. Importantly, computer support in medical diagnostics often requires algorithms that are appropriate for processing data expressing uncertainty and that can ensure high-quality diagnostics. To achieve this goal, a novel decision-making algorithm is used that employs interval entropy measures based on the theory of interval-valued fuzzy sets. Such an approach enables one to take into account diagnostic uncertainty, express it exactly, and interpret it easily. Furthermore, the applied classification technique offers the possibility of a straightforward explanation of the diagnosis, which is a situation required by many physicians. The presented solution combines innovative technological approaches with practical user needs, fostering the development of more effective tools in mental health prevention.

## 1. Introduction

This article presents some important results regarding work on a computer system designed to support the analysis of screening tests for identifying the risk of depressive episode occurrence and preventing depression. The use of machine learning algorithms will optimize systemic screening support for depression prevention, making the proposed solution a practical and efficient tool in public health. The proposed system will be available to researchers as a platform for knowledge and experience exchange, as well as to medical practitioners, particularly primary care physicians, to support them in identifying their patients’ depression risk. It is worth emphasizing that detecting the initial symptoms of depression and implementing the appropriate therapy often prevent the development of the disease. Depression is an increasingly common disease in modern societies. Computer-aided diagnosis of depression is the subject of various studies. For example, applying deep learning to natural language processing and using it to analyze social media posts for the diagnosis of depression has great potential [1,2]. However, more can and should still be done not only to diagnose the existing disease but also to identify the risk of its future occurrence, which, as mentioned, allows for its prevention in many situations. In [3], a methodology for diagnosing the risk of depression was proposed. However, the issue of data privacy still was a problem, i.e., how to learn to indicate the risk of depression effectively with small datasets. The answer is an implementation of algorithms reaping the benefits of limited cooperation, i.e., from exchanging only information about certain parameters of classification/models. This aspect is addressed in this work. One of the main priorities of the system is the protection of the privacy of data taken from various sources through the use of federated learning techniques [4,5]. The occurrence of this need is common, especially in smaller medical centers where the collected data are not sufficient to successfully use AI techniques to support diagnosis (in this case, depression) due to their small sizes and various internal defects. Some medical data are survey-based, and thus burdened with uncertainty resulting from the specificity of the human mind. For example, individuals may interpret and describe the same situation differently depending on their mood or well-being at various points in time. As a result, the decision-making process based on them is also subject to uncertainty. That is why using advanced methods to present and handle uncertainty is a key element of the methodology adopted in this work. A distinctive feature of the described research is the implementation of a nonstandard decision-making algorithm that integrates interval entropy measures from an epistemic and ontic perspective, based on the theory of interval-valued fuzzy sets (IVFSs) [6,7].

Thus, this paper focuses on two key aspects of modern decision support systems: learning from distributed but federated data sources and modeling various forms of uncertainty. We discuss the following:

1. A federated technique for generating a global decision as a method that can respect uncertainty and provide support in cases with the problem of incomplete data (in the form of interval-valued fuzzy sets) and different local models;
2. Collective decision-making enhanced the effectiveness of local models in early diagnostic systems for depression. These models were developed using a new method that incorporates entropy measures from an epistemic perspective. In this work, we broaden the concept of entropy by introducing new definitions of interval entropy, viewed through an epistemic lens. We also investigate their potential applications, particularly highlighting their usefulness in different areas of data analysis.

## 2. Related Works

Depression is an increasingly prevalent condition in modern societies. According to data from the World Health Organization (WHO), an estimated 3.8% of the adult population is affected by depression, which translates to approximately 280 million people worldwide. In cases of moderate or severe depressive episodes, life-threatening situations may arise. In the most severe cases, depression can lead to suicide, with over 700,000 people dying by suicide globally each year. Suicide is the fourth leading cause of death among individuals aged 15–29 [8]. Current methods for diagnosing early-stage depression often rely on diagnostic tools that serve as support for psychiatrists. These include various assessment scales and tests such as the Beck Depression Inventory, Hamilton Depression Rating Scale, PHQ-9, QIDS-SR, HDRS, GDS, CES-D, and CESD-R [9,10]. One of the most recent tools, the CESD-R, was developed in 2004 by William Eaton et al. as an updated version of the CES-D scale [11]. This scale is among the most widely used instruments in psychiatric epidemiology. The CESD-R is a self-report tool in which individuals respond to 20 statements describing their potential well-being or behaviors. Respondents choose from five possible answers, ranging from 0 (not at all or less than one day) to 4 (nearly every day for two weeks). The total score ranges from 0 to 80 points. According to the authors, a score of 16 points or higher may indicate a concerning result, suggesting the need for psychiatric or psychological consultation [9].

The 16-point threshold is a topic of debate in the medical community. Therefore, in this article, we utilize the recently proposed and more sensitive test SenDD [3] for machine learning, which evaluates the risk of depressive episodes more rigorously than existing methods in the literature. Moreover, the presented approach prioritizes data privacy (using diverse datasets) and is designed to accommodate imprecise data sources, including those with missing values. Federated learning addresses privacy concerns by enabling collaborative algorithm training without the necessity of exchanging sensitive data, thus tackling key challenges in data governance and privacy [4,12,13]. Unlike distributed learning within data center environments or traditional methods of processing private data, federated learning presents unique challenges, including communication efficiency, heterogeneity, and privacy. This paradigm has been extensively studied in works such as [4,5,14], and we investigate its potential application in diagnosing depression. The second key issue addressed in this work is the problem of missing data. To tackle this, the theory of interval-valued fuzzy sets demonstrates itself as an effective tool for classifying data with uncertainty. Interval-valued fuzzy sets [6,7] offer flexibility in defining the degree to which elements belong to a given set concept [15]. Researchers have introduced a variety of operators and measures for interval-valued fuzzy sets. These include aggregation measures such as implication, entropy, or similarity, as well as containment measures (precedence index). These have been applied in decision-support processes [16,17,18,19,20]. Representing uncertainty is crucial for decision-making in the case of incomplete information or imprecise data. The selection of an appropriate representation method depends on the problem’s context and the characteristics of the available data. In interval-valued fuzzy sets, uncertainty is managed by representing attribute values as intervals. This paper adopts an epistemic approach, where attribute or measurement values for individual objects are defined within the bounds of intervals. The literature contains several approaches for modeling uncertainty and imprecision to enable learning from incomplete or ambiguous data and classifying them, as explored in studies such as [21,22,23]. However, there is still a need for more effective methods to represent uncertainty. In this work, we propose interval entropy measures based on the concepts of possibility and necessity. To this end, we introduce various comparability measures to construct interval entropy measures that are either more restrictive or general compared to classical approaches. The proposed system integrates a federated machine learning algorithm with a novel diagnostic test to support preventive diagnostics particularly when the input data are incomplete.

## 3. Representation of Uncertainty by Interval-Valued Fuzzy Set Theory

The prevalent presence of uncertainty in real-life datasets constitutes the primary motivation for the research described in this paper. The direction and methods of the research, apart from enhancing federated learning, stem from many challenges of decision-making support, particularly in medical practice. Uncertainty or imprecision finds an effective representation through interval-valued fuzzy sets, as evidenced by numerous practical applications. The concept of interval-valued fuzzy sets is based on the understanding that uncertainty can arise from two distinct sources: epistemic and ontic. In the epistemic sense, which is exploited in this paper, uncertainty is attributed to the lack of precise knowledge, where the interval represents limit values encompassing a single desired value.

Application of IVFS to various real-world problems, such as pattern recognition, medical diagnosis, decision-making, and image thresholding, often involves leveraging critical measures like distances, inclusion, equivalence, similarity, or entropy within interval-valued fuzzy sets, as discussed in works such as [24,25,26,27].

Entropy measures, in particular, are a focal point of our research. We introduce a novel approach to defining these measures, specifically aimed at modeling uncertainty. Furthermore, these newly proposed entropy measures play a pivotal role as a core component of the innovative diagnostic system we have developed.

### 3.1. Orders in the Interval Setting

A crucial object in the interval-valued fuzzy set theory is a set $L^{I}=\left\{\left[\underline{\zeta },\bar{\zeta }\right]:\;\underline{\zeta },\bar{\zeta }\in \left[0,1\right],\;\underline{\zeta }⩽\bar{\zeta }\right\}$ which denotes a family of all subintervals of the unit interval. In the articles by Zadeh [7], Sambuc [6], Turksen [28], and Gorzalczany [29], an **interval-valued fuzzy set** (IVFS) *Z* in *X* (X≠∅) is defined as a mapping $Z:X\to L^{I}$ such that for each x∈X, the interval $Z\left(x\right)=[\underline{Z}\left(x\right),\bar{Z}\left(x\right)]$ expresses the degree of membership of an element *x* to *Z*. We assume that the considered universe of discourse will be finite—that is, $X=\{x_{1},\ldots ,x_{n}\}$. Further, the family of all interval-valued fuzzy sets in *X* will be denoted by IVFS(X). All elements $\left[\underline{\zeta },\bar{\zeta }\right]$ of $L^{I}$ such that $\underline{\zeta }=\bar{\zeta }$ are called crisp.

What is more, we may denote Z∈IVFS(X) as $Z=\left\{\langle x,Z\left(x\right)\rangle :x\in X,\;Z:X\to L^{I}\right\}$. The interval-valued fuzzy set is a straightforward generalization of a classical fuzzy set described by $\left\{\langle x,Z(x)\rangle :x\in X,\;Z:X\to [0,1]\right\}$. Indeed, for fuzzy sets, the membership of each element *x* is always a precisely given real number. On the other hand, the degree of membership of an element *x* to the interval-valued fuzzy set (perceived from the epistemic point of view—see [30]) is not precise: we know only its upper and lower bounds. This is why interval-valued fuzzy sets appear to be very useful for our considerations.

#### 3.1.1. Partial and Linear Orders

The best-known partial order in $L^{I}$ is defined as follows:

(1) $$\left[\underline{\zeta },\bar{\zeta }\right]\leq_{2}\left[\underline{\eta },\bar{\eta }\right]\;\Leftrightarrow \;\underline{\zeta }\leq \underline{\eta },\;\bar{\zeta }\leq \bar{\eta }.$$

with the joint and meet operations defined in $L^{I}$, respectively, as

$$\left[\underline{\zeta },\bar{\zeta }\right]\vee \left[\underline{\eta },\bar{\eta }\right]=\left[\max\left(\underline{\zeta },\underline{\eta }\right),\max\left(\bar{\zeta },\bar{\eta }\right)\right],\;\;\left[\underline{\zeta },\bar{\zeta }\right]\wedge \left[\underline{\eta },\bar{\eta }\right]=\left[\min\left(\underline{\zeta },\underline{\eta }\right),\min\left(\bar{\zeta },\bar{\eta }\right)\right].$$

 These create the structure ($L^{I},\vee ,\wedge$), which is a complete lattice, where $1_{L^{I}}=\left[1,1\right]$ and $0_{L^{I}}=\left[0,0\right]$ are the greatest and the smallest element of $\left(L^{I},{⩽}_{2}\right)$, respectively.

In many real-life problems, we need a linear order to compare any two intervals, so we are interested in extending the partial order ${⩽}_{2}$ to a linear one. We call this relation an admissible order as in the source paper [31], from which Definition 1 and Proposition 1 come.

**Definition** **1.**  *An order $\leq_{Adm}$ in $L^{I}$ is called **admissible linear** if*

*1.*  *$\leq_{Adm}$ is linear in $L^{I}$;*
*2.*  *For all $\zeta ,\eta \in L^{I}$$\zeta \leq_{Adm}\eta$ whenever $\zeta {⩽}_{2}\eta$.*

**Proposition** **1.**  *Let $\Psi ,\Upsilon :{\left[0,1\right]}^{2}\to \left[0,1\right]$ be two continuous aggregation functions such that for all $\zeta =\left[\underline{\zeta },\bar{\zeta }\right],\eta =\left[\underline{\eta },\bar{\eta }\right]\in L^{I}$, the equalities $\Psi \left(\underline{\zeta },\bar{\zeta }\right)=\Psi \left(\underline{\eta },\bar{\eta }\right)$ and $\Upsilon \left(\underline{\zeta },\bar{\zeta }\right)=\Upsilon \left(\underline{\eta },\bar{\eta }\right)$ hold if and only if ζ=η. If the order ${⩽}_{\Psi ,\Upsilon }$ on $L^{I}$ is defined by*

(2) $$\zeta {⩽}_{\Psi ,\Upsilon }\eta \Leftrightarrow \Psi \left(\underline{\zeta },\bar{\zeta }\right)<\Psi \left(\underline{\eta },\bar{\eta }\right)\;or\;\left(\Psi \left(\underline{\zeta },\bar{\zeta }\right)=\Psi \left(\underline{\eta },\bar{\eta }\right)\;and\;\Upsilon \left(\underline{\zeta },\bar{\zeta }\right)⩽\Upsilon \left(\underline{\eta },\bar{\eta }\right)\right),$$

*then ${⩽}_{\Psi ,\Upsilon }$ is admissible.*

Admissible orders were studied, e.g., in [32,33]. A construction of admissible linear orders based on aggregation functions was explored (increasing operation $A:{\left[0,1\right]}^{2}\to \left[0,1\right]$ such that A(0,0)=0 and A(1,1)=1 [34]).

#### 3.1.2. Possibility and Necessity Issue

We will now discuss alternative definitions of orders on $L^{I}$. We observe structures with possible and necessary comparability relations (called “interval orders” in the sense used in the papers of Fishburn in 1970–1985 or Fodor and Roubens (1994)) suitable to the epistemic issue, which follow an intuitive approach to many real-life problems. Properties of the presented below comparability relations were also considered and partially described in [35] or [36].

##### Necessary Relation

We define the following case of comparability of intervals restricted to the partial or linear order, i.e., necessary relation, which we may interpret as conjunctive (ontic) relation. We say that one interval contains a collection of true values of each variable smaller than or equal to all true values from the second interval.

(3) $$\zeta \leq_{nec}\eta \Leftrightarrow \bar{\zeta }\leq \underline{\eta },$$

where $\zeta ,\eta \in L^{I}.$ It can be interpreted as a satisfied relation for disjoint collections of values. We observe that in $L^{I}$, the relation $\leq_{nec}$ is antisymmetric, is transitive, and has the Ferrers property [35].

##### Possible Relation

This relation describes a more general situation, which we may write as follows:

(4) $$\zeta \leq_{pos}\eta \Leftrightarrow \underline{\zeta }\leq \bar{\eta },$$

where $\zeta ,\eta \in L^{I}.$

Notice that the relation $\leq_{pos}$ is more appropriate for the epistemic (disjunctive) setting of the intervals. So, if $\left[\underline{\zeta },\bar{\zeta }\right]$ is an imprecise description of variable ζ and $\left[\underline{\eta },\bar{\eta }\right]$ is an imprecise description of variable η, then $\left[\underline{\zeta },\bar{\zeta }\right]\leq_{pos}\left[\underline{\eta },\bar{\eta }\right]$ means that it is possible that the true value of ζ is smaller than or equal to the true value of η. Thus, the relation $\leq_{pos}$ can be interpreted straightforwardly [37].

### 3.2. Aggregation Functions

Now, we recall the concept of an aggregation function in $L^{I}$. We consider aggregation with respect to both relations ${⩽}_{2}$ and ${⩽}_{Adm}$. Later, we use the notation ≤ both for the partial or admissible linear order, with $0_{L^{I}}=\left[0,0\right]$ and $1_{L^{I}}=\left[1,1\right]$ as a minimal and maximal element of $L^{I}$, respectively. By replacing the monotonicity condition, the natural order ≤, with the admissible linear orders or relations $\leq_{pos}$ and $\leq_{nec}$, new types of aggregation functions are obtained [20] and denoted by $A_{pos}$, $A_{nec}$, respectively. Therefore, we use the concepts of aggregation consistent with the mentioned orders (similar to [20,33,38,39]) as follows:

**Definition** **2.**  *An operation $A:{\left(L^{I}\right)}^{n}\to L^{I}$, where n⩾2, is called an  **interval-valued aggregation function** if it is increasing with respect to the order ≤ (partial or admissible linear or possible or necessary), i.e., for all $\zeta_{i},\eta_{i}\in L^{I}$:*

(5) $$\zeta_{i}\leq \eta_{i}\Rightarrow A\left(\zeta_{1},\ldots ,\zeta_{n}\right)\leq A\left(\eta_{1},\ldots ,\eta_{n}\right)$$

$$and\;\;\;A\left(\underset{n\times }{\underset{︸}{0_{L^{I}},\ldots ,0_{L^{I}}}}\right)=0_{L^{I}},\;\;A\left(\underset{n\times }{\underset{︸}{1_{L^{I}},\ldots ,1_{L^{I}}}}\right)=1_{L^{I}}.$$

Sometimes, to shorten the notation, we write $A_{i=1}^{n}\left(\zeta_{i}\right)$ instead of $A(\zeta_{1},\ldots ,\zeta_{n})$. This is especially useful when the aggregated objects cannot be denoted concisely.

**Example** **1.**  *The following operations represent classic, possible (belonging to the set of pos-aggregation functions—$A_{pos}$), or necessary (belonging to the set of nec-aggregation functions—$A_{nec}$) aggregation functions:*

$$A_{mean}\left(\left[\underline{\zeta },\bar{\zeta }\right],\left[\underline{\eta },\bar{\eta }\right]\right)=\begin{array}{c} \left[\frac{\underline{\zeta }+\underline{\eta }}{2},\frac{\bar{\zeta }+\bar{\eta }}{2}\right] \end{array},$$

$$A_{pos}\left(\left[\underline{\zeta },\bar{\zeta }\right],\left[\underline{\eta },\bar{\eta }\right]\right)=\left\{\begin{array}{cc} 0_{L^{I}} & \zeta =\eta =0_{L^{I}}\\ {}[A\left(\underline{\zeta },\underline{\eta }\right),1], & \mathrm{otherwise}, \end{array}\right.$$

$$A_{nec}\left(\left[\underline{\zeta },\bar{\zeta }\right],\left[\underline{\eta },\bar{\eta }\right]\right)=\begin{array}{c} \left[\frac{\underline{\zeta }+\underline{\eta }}{2},\max\left(\frac{\underline{\zeta }+\bar{\eta }}{2},\frac{\bar{\zeta }+\underline{\eta }}{2}\right)\right] \end{array},$$

*where A is an aggregation function.*

### 3.3. Interval Entropy

We can now proceed to the concept of interval entropy. We propose various comparability measures used to define entropy, which may be more restrictive or general compared to the classical approach. Whatever kind of uncertainty there is (imprecision, vagueness, partial truth, measurement errors, uncertainty of interpretation/evaluation of features, and the like), one may be interested in measuring its extent. In probability theory, Shannon’s entropy [40] is commonly applied to quantify the average uncertainty of prediction in a random experiment. There exist also entropy-like measures in evidence theory (cf. [41]). De Luca and Termini [42] utilized entropy to evaluate the degree of fuzziness. Yager [43] proposed expressing the measure of uncertainty related to a fuzzy set by the distance between this fuzzy set and its complement. Later on, papers appeared in which generalizations of the entropy were defined for fuzzy sets, interval-valued fuzzy sets, and intuitionistic fuzzy sets (cf. [25,44,45]). Unfortunately, the proposed entropies still assumed scalar real values (from the unit interval), similar to fuzzy sets, and disregarding completely the fact that interval-valued fuzzy sets contain additional uncertainty connected with the inability or hesitance in the unambiguous determination of the membership function.

We are inspired by [46,47,48], which propose a definition of entropy based on partial or linear orders. In comparison with [47], we extend in this paper the definition of entropy by adding a fourth axiom, which is essential from the application point of view. It is an axiom of the stability of entropy with respect to negation *N* ($N:L^{I}\to L^{I}$ that is decreasing with respect to ≤ with $N\left(1_{L^{I}}\right)=0_{L^{I}}$ and $N\left(0_{L^{I}}\right)=1_{L^{I}}$ [49]). The presented definition proposed and studied in [50] also extends the definition of [48] by using the width of intervals (axiom e2), so uncertainty information is reflected more. But before that, we will introduce the following notation: for $e\in L^{I}$, such that N(e)=e, i.e., *e* is the equilibrium point, we denote by E∈IVFS(X) a mapping such that E(x)=e for all x∈X. Moreover, in this article, we develop the concept of entropy by introducing yet other (in the epistemic and ontic sense) definitions of interval entropy, and in the following sections, we examine their application potential, with particular emphasis on applications in broadly perceived data analysis.

**Definition** **3.**  *Let N be a strong (i.e., involutive and decreasing) interval negation with equilibrium point $e\in L^{I}$ closest to the point [0.5,0.5]. A function $E_{I}:\mathrm{IVFS}\left(X\right)\to L^{I}$ is an **interval entropy** on IVFS(X) with respect to the negation N and order ≤ if for A,B∈IVFS(X), the following hold:*

*(e1)*  *$E_{I}\left(S\right)=0_{L^{I}}$ iff S is crisp;*
*(e2)*  *$E_{I}\left(E\right)=\left[1-w\left(e\right),1\right]$, where w(e) is a width of interval e;*
*(e3)*  *$E_{I}\left(A\right)\leq E_{I}\left(B\right)$, if A(x)≤B(x)≤e or A(x)≥B(x)≥e for all x∈X;*
*(e4)*  *$E_{I}\left(A\right)=E_{I}\left(A_{N}\right)$, where $A_{N}\left(x\right)=N\left(A\left(x\right)\right)$ for all x∈X.*

The next theorems show how to apply a similarity measure and adequate negation function from each class (standard, as well as possible and necessary as in [3]) for constructing an interval entropy. In the classic case, we have the following method of interval entropy construction [50]:

**Proposition** **2.**  *Let $S_{std}$ be a similarity measure that satisfies conditions given in [50], where $A_{1}$ is an idempotent aggregation function. Then, a function $E_{I}:\mathrm{IVFS}\left(X\right)\to L^{I}$ defined as*

(6) $$E_{I}\left(A\right)=S_{std}\left(A,A_{N}\right)$$

*is the interval entropy with respect to an involutive interval negation N with equilibrium point e.*

**Example** **2.**  *If we use a similarity measure given in [50] with any precedence indicator considered there, we obtain the interval entropy measures that fulfill condition (e4) in Definition 3 for the standard interval negation $N\left(x\right)=\left[1-\bar{x},1-\underline{x}\right]$, where $x=[\underline{x},\bar{x}]$.*

Let us consider a new measure of possible entropy, also called an optimistic entropy measure that takes into account the comparability relation of intervals.

**Definition** **4.**  *Let $N_{pos}$ be a strong involutive negation of the possibility of the equilibrium point $e\in L^{I}$ ($N_{pos}\left(e\right)=e$). Function $E_{pos}:IVFS\left(X\right)\to L^{I}$ is the interval possible entropy on IVFS(X) with respect to the negation N if for A,B∈IVFS(X), the following hold:*

*(EP1)*  *$E_{pos}\left(A\right)=0_{L^{I}}$, if A is a crisp set;*
*(EP2)*  *$E_{pos}\left(E\right)=\left[1-w\left(e\right),1\right]$, where w(e) is the width of interval e;*
*(EP3)*  *$E_{pos}\left(A\right)\leq_{pos}E_{pos}\left(B\right)$, if $A\left(x\right)\leq_{pos}B\left(x\right)\leq_{pos}e$ or $A\left(x\right)\geq_{pos}B\left(x\right)\geq_{pos}e$ for all x∈X.*

The method of constructing the interval optimistic (possible) entropy based on the possible similarity measure is as follows:

**Proposition** **3.**  *Let $S_{pos}$ be a possible similarity measure. Then, the function $E:IVFS\left(X\right)\to L^{I}$*

(7) $$E_{pos}\left(A\right)=S_{pos}\left(A,A_{N_{pos}}\right)$$

*is the interval possible entropy with respect to $N_{pos}$ (involute interval possible negation with equilibrium point e).*

**Proof.**  If *A* is a crisp set, then we directly obtain $E_{pos}\left(A\right)=0_{L^{I}}.$ We can obtain (EP2) directly by the idempotency of the similarity measure and the idempotency of the aggregation function A.

If $A\leq_{pos}B\leq_{pos}E$, then $A\leq_{pos}B\leq_{pos}E\leq_{pos}B_{N_{pos}}\leq_{pos}A_{N_{pos}}$ and, consequently, from the monotonicity of the similarity measure, we have
  $$E_{pos}\left(A\right)=S_{pos}\left(A,A_{N_{pos}}\right)\leq_{pos}S_{pos}\left(A,B_{N_{pos}}\right)\leq_{pos}S_{pos}\left(B,B_{N_{pos}}\right)=E_{pos}\left(B\right).$$

In a similar way and by the symmetry of the similarity measure, we can prove the case $A\geq_{pos}B\geq_{pos}E$, which ends the proof of (EP3) and Proposition 3.    □

**Example** **3.** *If we use the possibility similarity measure from [3], we then obtain an entropy measure that satisfies Equation (7) with respect to $N\left(x\right)=\left[1-\bar{x},1-\underline{x}\right]$ for*

$$S_{pos}\left(A,B\right)=A_{i=1}^{n}B\left({\mathrm{Prec}}_{pos}\left(A\left(x_{i}\right),B\left(x_{i}\right)\right),{\mathrm{Prec}}_{pos}\left(B\left(x_{i}\right),A\left(x_{i}\right)\right)\right),$$

*where $A=A_{mean},B=\wedge$ and ${\mathrm{Prec}}_{pos}$ fulfills*

$${\mathrm{Prec}}_{A}\left(a,b\right)=\left\{\begin{array}{cc} {}[1-w(a),1], & \mathrm{if}\;a=b,\\ 1_{L^{I}}, & \mathrm{if}\;a{<}_{pos}b,\\ A_{pos}\left(N_{pos}\left(a\right),b\right), & \mathrm{else}. \end{array}\right.$$

Another class of entropy is the necessary entropy measure, which uses the necessary relation when comparing intervals.

**Definition** **5.**  *Let $N_{nec}$ be a strong (involute) interval necessary negation with an equilibrium point $e\in L^{I}$. The function $E_{nec}:IVFS\left(X\right)\to L^{I}$ is the interval necessary entropy on IVFS(X) with respect to the negation of $N_{nec}$ if for A,B∈IVFS(X),*

*(EN1)*  *$E_{nec}\left(A\right)=0_{L^{I}}$, if A is a crisp set;*
*(EN2)*  *$E_{nec}\left(E\right)=\left[1-w\left(e\right),1\right]$;*
*(EN3)*  *$E_{nec}\left(A\right)\leq E_{nec}\left(B\right)$, if $A\left(x\right)\leq_{nec}B\left(x\right)\leq_{nec}e$ or $A\left(x\right)\geq_{nec}B\left(x\right)\geq_{nec}e$ for all x∈X.*

Analogously to possible entropy, based on the necessary similarity measure, we can obtain the necessary entropy:

**Proposition** **4.**  *Let $S_{nec}$ be the necessary similarity measure. Then, the function $E_{nec}:IVFS\left(X\right)\to L^{I}$*

(8) $$E_{nec}\left(A\right)=S_{nec}\left(A,A_{N_{nec}}\right)$$

*is the interval necessary entropy with respect to $N_{nec}$ (involutional interval necessary negation with equilibrium point e).*

**Proof.**  If *A* is a crisp set, then (cf. Example 3)

$$\left\{{\mathrm{Prec}}_{nec}\left(A\left(x_{i}\right),B\left(x_{i}\right)\right),{\mathrm{Prec}}_{nec}\left(B\left(x_{i}\right),A\left(x_{i}\right)\right)\right\}\in \left\{0_{L^{I}},1_{L^{I}}\right\}$$

and because $B_{nec}$ has a neutral element $1_{L^{I}}$, we obtain $E_{nec}\left(A\right)=0_{L^{I}}.$

(EN2) can be directly obtained from the idempotency similarity measure.
If $A\leq_{nec}B\leq_{nec}E$, then $A\leq_{nec}B\leq_{nec}E\leq_{nec}B_{N_{nec}}\leq_{nec}A_{N_{nec}}$ and by the isotonicity of the necessary similarity measure we have
  $$E_{nec}\left(A\right)=S_{nec}\left(A,A_{N_{nec}}\right)\leq_{nec}S_{nec}\left(A,B_{N_{nec}}\right)\leq_{nec}S_{nec}\left(B,B_{N_{nec}}\right)=E_{nec}\left(B\right).$$
In a similar way, by the symmetry of the similarity measure, we can prove the following case: $A\geq_{nec}B\geq_{nec}E$. This ends the proof of (EN3) and this proposition.    □

**Example** **4.**  *If we use the necessary similarity measures from [3], then we obtain an entropy measure that satisfies Equation (8) with respect to $N\left(x\right)=\left[1-\bar{x},1-\underline{x}\right]$.*

## 4. Problem and Idea of Solution and Methodology

In this work, we support the problem of early diagnosis/prevention of depression, especially when a given medical center has few or incomplete data.

We investigate such situations when different data sources (medical centers supporting the diagnosis of depression prevention) have different datasets, not excluding imbalanced data (NON-IID data, i.e., Non-Independently and Non-Identically Distributed Data), and for privacy protection reasons (restrictive privacy regulations applicable to the creation of datasets) cannot make their data available to other centers. Alone, they have low diagnostic effectiveness. For such situations, we propose incorporating the idea of federated decision-making based on models obtained independently from separated data sources.

### 4.1. Dataset Description

The database on which the research was carried out in this work consists of answers to two diagnostic tests, (CESD-R (The Center for Epidemiologic Studies Depression Revised Scale) and SenDD (Sensitive Depression Diagnosis)), received by the CAWI technique. The same 750 students were respondents to each test. This collection will be expanded in the future, but as a cross-section of the student environment, it was a good basis for research.

The SenDD test includes 22 questions (condition attributes) plus a binary diagnosis (decision attribute). Condition attributes consist of 20 basic questions (attributes 1–20) and 2 verification questions (attributes 21–22) [3]. The answers to each question are as follows: 

- Definitely yes: 4 points;
- Rather like this: 3 points;
- It’s hard to say: 2 points;
- Probably not: 1 point;
- Definitely not: 0 points.

All twenty basic questions follow the pattern “the more, the worse”, while two verification questions follow the pattern “the more, the better”.

The dataset was constructed in two steps:

1. Answers to questions from the SenDD test (questions 1–22)
2. Decisions created in the following way:
  – For answers to questions 1–20, decisions obtained by the same respondent in the CESD-R test were assigned, a so-called initial (assuming a two-class representation of the decision): depression or no depression.
  – Then, using the SENSDEPR [3] algorithm, decisions that were on the borderline of decisions between classes were evaluated using verification questions and modified to classes: depression threat (includes cases of depression) and no depression.

The dataset used in the experiments described in this paper consists of 750 records (objects) with 501 “0” (no depression) values and 249 “1” (depression or subliminal depression) values as decision values. Moreover, it includes missing values on the level of approximately 5%. The dataset is described in more detail in [3].

The effectiveness in terms of obtaining greater sensitivity of the SenDD test compared to other datasets, as well as the method presented in Algorithm 1, was confirmed by using the CESD-R test after the initial calculation of the diagnosis (modified to binary: sick “1” or healthy “0” ). Verification was realized in two-stage studies (repeated every 4 weeks on the same group of subjects for both tests) and we verified the SenDD test for its more sensitive diagnostics—i.e., earlier (by 4 weeks) indication of depression. This is an important piece of information regarding more sensitive tests (20% of respondents confirmed depression 4 weeks later with the CESD-R test) [3].
**Algorithm 1:** Diagnosing danger of depression
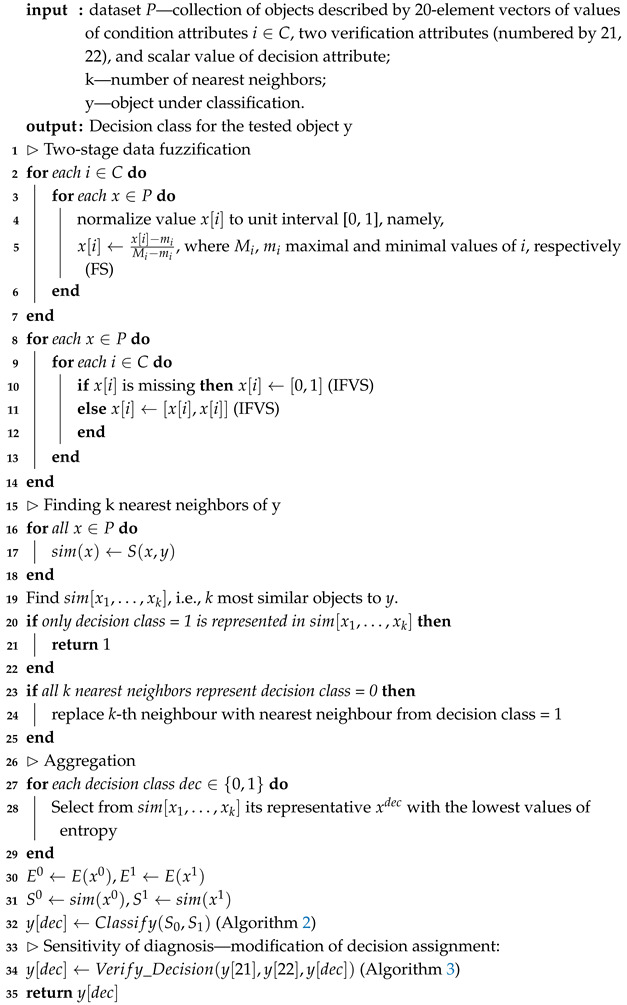


### 4.2. The Procedure of Depression Prediction

The procedure proposed below takes into account all the research challenges we set: i.e., maintaining privacy and handling imperfections in data, such as uncertainty or non-balanced data. The procedure consists of the following three stages:

1. Algorithm 1 (included also Algorithms 2 and 3)—which learns depression prediction in the local mode, i.e., individually for each local data in the form of SenDD test answers. The algorithm utilizes interval entropy and similarity measures. Its role is analogous to learning individual classifiers of ensemble classifiers;
2. Algorithm 4—which generates collective and optimal decisions for several NON-IID data. It is a kind of classification conflict resolution;
3. Algorithm 5 (“Decision Rule”)—which accepts or rejects changes in the decision value suggested by Algorithm 4.

We will discuss all three stages below.
**Algorithm 2:** Procedure Classify (interval $dec_{0}$, interval $dec_{1}$)

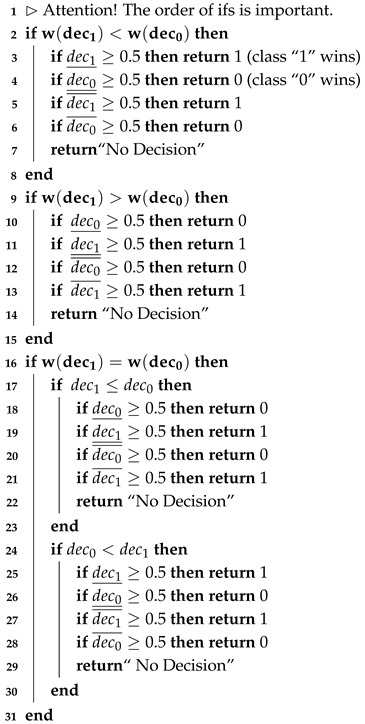


**Algorithm 3:** Procedure Verify_Decision (interval $val_{1}$, interval $val_{2}$, interval dec)


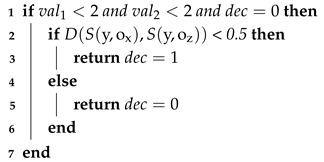



**Algorithm 4:** Federated system for diagnosing risk of depression

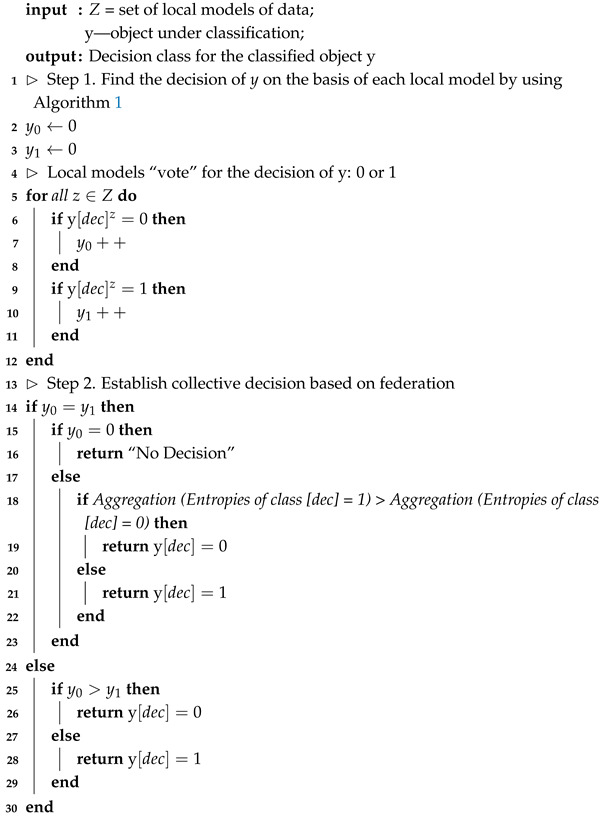



**Algorithm 5:** Procedure Decision_Rule (measure *EF∈{ACC, SENS, SPEC, PREC}*, table of EF values for all datasets $z_{i}$ and federated model with final decision (FD))

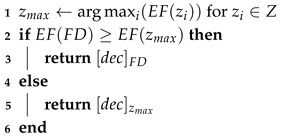



#### 4.2.1. New Algorithms for Diagnosis of Depression

Algorithm 1 for depression detection is based on the answers to the SenDD questionnaire. The proposed algorithm is an epistemic extension of the SENSDEPR algorithm [3]. Unlike the previous algorithm, we propose the use of entropy as a measure of assessing data uncertainty.

In the initial phase of the algorithm, we perform a two-stage fuzzification procedure on the input data values. This reduces each value to an interval from the $L^{I}$ family. In particular, each certain value *a* is normalized to the value b∈[0,1] and replaced by the interval [b,b]. Missing values are replaced by the interval [0,1].

The kNN algorithm is the basis of the approach proposed in Algorithm 1. The choice of kNN as the classification method used in the system was dictated, first of all, by the research conducted in [3,9,51,52], where kNN turned out to be one of the most effective methods in the problem of diagnosing depression. As a well-explained tool, it is the best choice for our system. By good explainability, we mean a process of searching for a diagnosis and its interpretation that is understandable by domain specialists. The technique we propose for searching for a diagnosis among objects with a similar distribution of features (similar answers) is analogous to the procedure used in medicine called “case study” and is accepted by medical communities. Additionally, taking into account imprecision through the use of IVFS provides a natural combination of machine learning and human thinking often associated with uncertainty. In the mentioned articles, we may observe comparing methods of classification with results obtained using other methods of machine learning and different psychiatric tests with new tests used in the diagnosis of depression. For example, in [51], one can observe an application of the kNN and Random Forest algorithms for diagnosing depression based on CESD-R test answers. In this application, the kNN algorithm classified better than Random Forest. The choice of test CESD-R as a source of decision values assigned to objects from the SenDD dataset was dictated by its efficiency in comparison with the following depression diagnosing tests: Beck, Hamilton Depression Rating Scale, PHQ-9, QIDS-SR, HDRS, GDS, and CES-D [3,9,10]. The obtained dataset in our research was used for testing a new, more effective, and sensitive diagnostic model based on the following:

- SenDD;
- Algorithms 1–4;
- Algorithm 5 (Decision Rule), which is explained in the next part of this paper.

The selection of nearest neighbors of a classified object is realized first by choosing *k* most similar (according to used similarity measure) objects from input data and next by taking the most similar representatives of each decision class present among k nearest neighbors. Interval values of similarity measures are the basis of the choice of the decision value for the object under classification. In particular, in the presented Algorithm 1, we apply the “selection method” based on the width of intervals, a critical point 0.5, and four types of order, namely, $\leq \;\in \;\{\leq_{2},\leq_{Adm},\leq_{pos},\leq_{nec}\}$.

Then, among the *k* most similar objects to *y* in each decision class (lines 16–19), we use the object entropy measure and select the one with the lowest uncertainty, i.e., the lowest entropy (lines 21–22 of the algorithm code). Thus, in the next step of the algorithm, for the two intervals obtained corresponding to each class, we use the following method leading to the decision-making, where for the classes “0” (no depression) and “1” (depression or subliminal depression), intervals are denoted as $d_{0}$ and $d_{1}$, respectively, in accordance with their connection with the neutral value [0.5,0.5]. In addition, the algorithm may refrain from making decisions “No Decision” (procedure Classify (Algorithm 2) in line 24). This, in turn, is subject to possible correction in the last part of the algorithm depending on the patient’s answers to the last two so-called verifying questions (attributes 21 and 22 with values less than 2 on a scale [0–4], which means a negative psychological situation for the respondent, according to expert assessment). In such a situation, for a primary diagnosis that does not indicate depression, a change is made (procedure Verify_Decision (Algorithm 3) on line 26).

The following symbols are used in Algorithms 1–3:

- x—element of input dataset P;
- x[i]—i-th coordinate of vector x;
- w(r)—width of interval r;
- $\underline{r}$, $\bar{r}$—left and right end of interval r;
- $o_{x}$—object which is the most similar to y;
- $o_{z}$—object which is the most similar to y with decision value “1”, i.e., $o_{z}\left[dec\right]=1$;
- $o_{x}\left[21\right],$$o_{x}\left[22\right]$—values of attributes 21, 22
  (the answers for verification questions 21 and 22) of object $o_{x}$;
- D—distance measure,
  $$D\left(\left[\underline{\zeta },\bar{\zeta }\right],\left[\underline{\eta },\bar{\eta }\right]\right)=\max(|\underline{\zeta }-\underline{\eta }|,|\bar{\zeta }-\bar{\eta }|);$$
- *E*—entropy measure constructed by methods of Propositions 6, 9, and 12, respectively, for classes standard, possible, and necessary ($E\in \{E_{std},E_{pos},E_{nec}\}$);
- *S*—similarity measure constructed by methods from [3,50]: $S\in \{S_{std},S_{pos},S_{nec}\}$ ($S_{pos}$ cf. Example 10).

#### 4.2.2. Federated Decision-Making

In Algorithm 4, which is the second step of the procedure for depression prediction, we present a method for generating the final decision—a global decision. It uses decisions obtained in Algorithm 1 based on data in each local set participating in the federation for a given object. The federated decision (final decision, Algorithm 5) is based on the idea of federated learning [4,5], where decisions obtained on local data have an impact on the final decision (provided that they do not spoil the effectiveness of the local model) based on the plurality voting technique. The proposed method additionally takes into account the use of the entropy measure in the case of an equal number of obtained local decisions for each class.

The **Decision Rule** procedure (Algorithm 5) suggests federated decision-making ${\left[dec\right]}_{FD}$ (Algorithm 4) only for local sets with local model efficiency lower than the collective decision designation in order to make a final decision (FD). The goal is to not worsen the quality of classification for all local datasets.

Let us recall the definitions of some of the coefficients used in Algorithm 5 and experiments:

- Accuracy: $ACC=\frac{TP+TN}{TP+TN+FP+FN}$;
- Specificity: $SPEC=\frac{TN}{TN+FP}$;
- Precision: $PREC=\frac{TP}{TP+FP}$;
- Sensitivity: $SENS=\frac{TP}{TP+FN}$.

Here, TP stands for the number of correctly classified positive cases, TN is the number of correctly classified negative cases, FP is the number of incorrectly classified negative cases, and FN is the number of incorrectly classified positive cases.

Proposing the use of the new entropy in the epistemic aspect in both of the new proposed algorithms (generating an individual decision in individual local sets and generating a federated decision) is a novelty presented in this paper and the effectiveness of this will be presented in the next section.

## 5. Experimental Results of Different Aspects of Real Problems

We demonstrate the effectiveness of the proposed algorithms using widely used measures of accuracy (ACC), sensitivity (SENS), specificity (SPEC), and precision (PREC).

We investigate the mentioned values of effectiveness obtained in the following scenarios:

1. Scenario 1: centralized model. The model was trained on a 70% input dataset and tested on a 30% input dataset. We compared the SENSDEPR algorithm [3] and Algorithm 1.
2. Scenario 2: local models. Due to biased data division (NON-IID data), the distribution of classes among clients was uneven. We created three local models from the input dataset. Client 1 received a balanced division of classes with a 50–50% split of decision classes. However, Clients 2 and 3 received an unbalanced distribution, with one having 80% cases of class 0 and the other having 80% cases of class 1. Local models were tested on a balanced 15% test set separated from the dataset before splitting into local sets.
3. Scenario 3: federated decision-making based on Algorithm 4. The method was used only for local models with lower effectiveness than the federation’s effectiveness (Algorithm 5).

**Figure 1:**
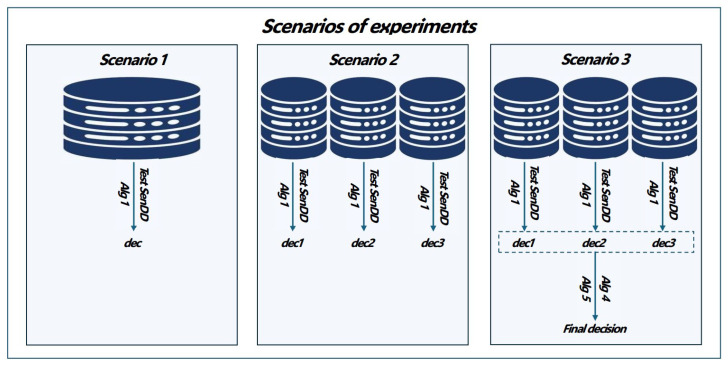
Experimental scenarios.

It is worth noting that during the experiments (in Figure 1), only one effectiveness measure was applied in the Decision Rule procedure (Algorithm 5). Namely, it was the accuracy measure. Despite this, values of other efficiency parameters (SENS, SPEC, PREC) were also recorded.

In each scenario, 10-fold cross-validation was used. In addition, various model parameters were considered:

- k∈{1,3,5,7};
- Operator aggregation functions, orders, and similarity were used to build adequate classes (standard/classic, possible, and necessary for different entropy measures).

We present the best results in Table 1, Table 2, Table 3 and Table 4.

Firstly, in Scenario 1, i.e., in the Baseline Model, where the dataset was complete and lacked any uncertainty (due to removing cases with missing values), the SENSDEPR algorithm described in [3] (which has also been compared with other methods of diagnosis given in the literature) is compared with Algorithm 1. The outputs are presented in Table 1 for optimal results obtained in the considered possible cases (possible similarity, entropy, and aggregation were used).

Experiments conducted according to scenarios 2 and 3 are presented separately for the three discussed classes of entropy measures: classic, possible, and necessary aspects. The results demonstrate that the federated model, corresponding to scenario 3, achieved higher efficiency compared to the other scenarios. We observed enhanced performance in key metrics such as accuracy, sensitivity, specificity, and precision. Improving sensitivity and specificity is particularly important for NON-IID data, especially for possible entropy measures used in Algorithm 1. A similar dependency arose for the centralized model when compared with the most effective similarity measure used in the SENSDEPR algorithm [3]. This observation is significant as it underscores the potential of the proposed algorithm in future studies of other real problems by using **possible measure similarity or entropy**, i.e., optimal measures from the point of view of representation uncertainty. We also present the best results of classification efficiency in the tables below when using accuracy as efficiency in the Decision Rule algorithm (Algorithm 5) (EF = ACC).

Firstly, in Table 2, we can see the performance of the model on NON-IID data in the classic case:

Now, in Table 3, we present the performance of the model on NON-IID data in the possible case:

Finally, we compare in Table 4 the above results with the performance of the model on NON-IID data in the necessary case:

In Table 1, Table 2, Table 3 and Table 4, we present the best results for each class for different parameters such as aggregations, order, and number of k (k=5) for similarities of objects and, finally, entropy measures. The best results were observed for **possible measures**, especially for $S_{pos}$ (from Example 3), which was also used in the construction of **possible entropy measures**. We see the greatest progress (we improved the efficiency of the classification of weak clients while not worsening the efficiency of strong partners (clients) of the federation) for key measures such as ACC and PREC, which is a priority for equally important problems such as people’s health and safety.

## 6. Summary and Future Plans

In this article, we demonstrated an actual application and the use of new definitions of entropy in the necessary, possible, and standard aspects of a modification of the diagnostic model based on a generalized kNN algorithm. We introduced entropies in the optimistic and pessimistic aspects of interval-valued fuzzy relations. The new algorithm creates a more sensitive diagnosis. We also proposed an ensemble decision-making method for small and NON-IID datasets.

The interval calculus approach proposed in the article turned out to be very effective. Intervals were used to represent the uncertainty of data, and the comparability measures related to the interval calculus take into account different epistemic, more or less restrictive (possible or necessary) approaches, as illustrated with the comparability measures considered—i.e., entropy measures tend to be more flexible due to uncertainty.

In particular, the new entropy measure proposed in this paper proved to be useful for improving the efficiency of the proposed algorithm for proposing decisions under conditions of small or faulty datasets and in cases dealing with data privacy problems.

Proposing the use of entropy in both algorithms (generating an individual decision in individual local sets and generating a federated decision) is a novel aspect in this paper.

The research we present is preliminary research. In the next stages, we plan to obtain consent for clinical trials from a relevant bioethics committee and expand the research in the following areas:

- Consideration of different screening periods to determine the sensitivity threshold of the test;
- Use of different methods of the imputation of missing data;
- Taking into account the influence of membership in different social groups on decisions (examination of the influence of social–psychological characteristics on decisions), different types of data, etc.

Moreover, in the future, we will continue tests on the correctness of the process of decision-making to tune algorithms to work in an online system for producing fast diagnoses. The created system will be used in screening selected risk groups, including school-age children, who increasingly exhibit mood disorders. The system for diagnosing risk of depression will be widely used among people of different age groups. Thus, the database will be expanded.

Realizing that the proposed system, especially the local classification of threats, is strongly related to the specificity of a given problem/disease entity, the problem of developing the indicated federated decision-making technique will be associated with the modification of the classification algorithm in relation to the new research problem.

The development of the methodology will take the following into account:

- Various data types, including categorical data. The database will be expanded with additional information about living conditions collected in interviews to propose a relationship between the diagnosis and the socioeconomic situation of respondents.
- Comparison with other methods used in the diagnosis of depression, as well as analysis of the potential use of other methods taking into account data noise.

## Figures and Tables

**Table 1:** Performance of algorithms with full data.

Scenarios	Dataset	ACC	SENS	SPEC	PREC
Scenario 1—Baseline—Algorithm SENSDEPR	All Data	0.97	**0.81**	0.984	**0.981**
Scenario 1—Baseline—Algorithm 1	All Data	**0.988**	0.785	**0.989**	0.865

**Table 2:** Performance on NON-IID data in classic case.

Scenarios	Dataset	ACC	SENS	SPEC	PREC
Scenario 2	Client 1	**0.764**	0.781	0.685	0.744
–Local Models	Client 2	0.653	0.58	0.904	0.599
	Client 3	0.616	0.973	0.646	0.616
Scenario 3—Federated Decision-Making (Algorithm 4)		0.723	0.715	0.735	0.732
Decision Rule (Algorithm 5)		**0.764**	0.781	0.685	0.744

**Table 3:** Performance on NON-IID data in possible case.

Scenarios	Dataset	ACC	SENS	SPEC	PREC
Scenario 2	Client 1	**0.887**	0.887	0.667	0.854
–Local Models	Client 2	0.871	0.488	0.959	0.654
	Client 3	0.642	0.987	0.354	0.642
Scenario 3—Federated Decision-Making (Algorithm 4)		0.7125	0.635	0.79	0.769
Decision Rule (Algorithm 5)		**0.887**	0.887	0.667	0.854

**Table 4:** Performance on NON-IID data in necessary case.

Scenarios	Dataset	ACC	SENS	SPEC	PREC
Scenario 2	Client 1	0.695	0.67	0.72	0.717
–Local Models	Client 2	**0.746**	0.467	0.895	0.653
	Client 3	0.597	0.895	0.573	0.572
Scenario 3—Federated Decision-Making (Algorithm 4)		0.701	0.685	0.7175	0.716
Decision Rule (Algorithm 5)		**0.746**	0.467	0.895	0.653

## Data Availability

Anonymized data are available at the following link: https://tiny.pl/92s9g (accessed on 25 January 2025).
